# Electrochemical Detection of Alpha-Fetoprotein Based on Black Phosphorus Nanosheets Modification with Iron Ions

**DOI:** 10.3390/mi13050673

**Published:** 2022-04-26

**Authors:** Yiyan Chen, Xiaoping Chen, Jianwei Lin, Yafeng Zhuang, Zhizhong Han, Jinghua Chen

**Affiliations:** 1 School of Pharmacy, Fujian Medical University, Fuzhou 350122, China; cyy20201323@163.com (Y.C.); cuacuxsoe@163.com (X.C.); a15260712344@163.com (J.L.); 18760367357@139.com (Y.Z.); cjh_huaxue@fjmu.edu.cn (J.C.); 2 Fujian Key Laboratory of Drug Target Discovery and Structural and Functional Research, Fuzhou 350122, China

**Keywords:** black phosphorus nanosheets, Fe^3+^, electrochemical immunosensors, alpha-fetoprotein

## Abstract

Black phosphorus nanosheets (BPNSs) were synthesized with liquid exfoliation combined with the ultrasonic method and loaded with Fe^3+^ by simply mixing. The morphology, structure and electrochemical properties of the synthesized Fe^3+^/BPNSs were characterized by transmission electron microscopy (TEM), atomic force microscopy (AFM), Raman spectroscopy, X-ray photoelectron spectroscopy (XPS) and cyclic voltammetry (CV), etc. The load of Fe^3+^ can improve the electrochemical performance of BPNSs and enhance the sensitivity of the detection. Additionally, Fe^3+^/BPNSs display good biocompatibility. In this study, immunosensors based on Fe^3+^/BPNSs were constructed to detect alpha-fetoprotein (AFP). The detection is due to the specific binding between the AFP antigen and antibody on the surface of the immunosensors, which can reduce the current response of Fe^3+^/BPNSs. The immunosensors have a good linear relationship in the range of 0.005 ng·mL^−1^ to 50 ng·mL^−1^, and the detection limit is 1.2 pg·mL^−1^. The results show that surface modification with metal ions is a simple and effective way to improve the electrochemical properties of BPNSs, which will broaden the prospects for the future application of BPNSs in the electrochemical field.

## 1. Introduction

Alpha fetoprotein (AFP) is a type of glycoprotein that is closely related to the occurrence of liver cancer and a variety of tumors, and can be used as a serum marker of liver cancer in the clinical setting [1]. When the concentration of AFP in the body exceeds 30 ng·mL^−1^, the presence of a malignant tumor can be considered. Therefore, it is very necessary to establish a rapid and sensitive method for the detection of AFP. In recent years, there have been many methods for detecting AFP—for example, electrochemiluminescence [2,3], fluorescence immunoassay [4,5], chemiluminescence immunoassay [6] and enzyme-linked immunoassay [7]. Electrochemical immunosensors have been widely used in the detection of AFP in recent years due to their high sensitivity, good selectivity and simple instrumentation, which have advantages in the detection of AFP [8,9]. Compared with labeled immunosensors, unlabeled electrochemical immunosensors have attracted more and more attention because of their advantages of simple and convenient preparation [10,11,12].

Since 1914, the anisotropy of black phosphorus (BP) in electrical, mechanical, optical and chemical properties has given it fascinating properties and great application potential. Black phosphorus nanosheets (BPNSs), as a two-dimensional material of BP, were first reported in 2014 and have advantages in terms of ultra-high specific surface area and anisotropic conductivity [13]. At present, the preparation methods of BPNSs include mechanical exfoliation [14], ultrasonic liquid-phase stripping [15], electrochemical stripping, pulsed laser deposition, [16], etc. The ultrasonic liquid stripping method is simple and low-cost, and the average size of the stripping product can be controlled by adjusting the ultrasonic frequency.

Compared with other two-dimensional materials, such as graphene and MoS_2_, BPNSs have higher carrier mobility and a better optical response [17], which determine their wide applications in fluorescence sensing [18], photoelectronics and other fields. The easy oxidation of BPNSs in air has led to their lesser use in electrochemistry. Many researchers have improved the properties of BPNSs by surface modification or passivation—for example, the diazotization of nucleophiles linked to BPNSs [19], nanoparticle loading [20,21] and metal cation–π interactions [22]. Lone pair electrons in BPNSs can easily react with oxygen to form P_x_O_y_. If the lone pair electrons in BPNSs are occupied by cations, the oxidation of BPNSs will be prevented. Among the cations, Fe^3+^ has a greater positive charge. The greater positive charge causes a greater change in the electron transport performance of BPNSs, thus enhancing the electrochemical performance [20].

In this paper, BPNSs were prepared by the ultrasonic liquid-phase stripping method. The electrochemical performance of BPNSs was effectively improved by simply loading Fe^3+^ on their surface, and then the modified composites were used in the electrochemical detection of AFP. Because the protein is insulated, the current reduces when the AFP antibody and antigen are added. The current change is proportional to the concentration of AFP on the electrode surface. Then, electrochemical immunosensors based on Fe^3+^/BPNSs were constructed to detect AFP. The immunosensors display good electrochemical properties, which expand the electrochemical application of BPNSs.

## 2. Experimental

### 2.1. Reagents and Instruments

Iron(III) chloride (FeCl_3_) and N-methylpyrrolidone (NMP) were purchased from Shanghai Macklin Biochemical Co., Ltd. (Shanghai, China). Black phosphorus crystal powder was bought from Nanjing XFNANO Technology Co., Ltd. (Nanjing, China). Phosphate-buffered saline (PBS) was obtained from Cytiva Co., Ltd. (Marlborough, MA, USA). Bovine serum protein (BSA) was purchased from Sangon Biotech Co., Ltd. (Shanghai, China). Alpha-fetoprotein (AFP) antibody was purchased from Zhengzhou Cell To Antibody&Antigen Biotechnology. Co., Ltd. (Zhengzhou, China).

The UV–vis absorption spectra were recorded on a Thermo Fisher GENESYS-150 Spectrophotometer (Waltham, MA, USA). The fluorescent spectra were obtained by a F96Pro-type fluorescent photometer (Lengguang, Shanghai, China). The transmission electron microscopy (TEM) images were taken on a JEM 2100F transmission electron microscope (Tokyo, Japan). The Fourier infrared spectrum (FTIR) was recorded by a Thermo Fisher Scientific Nicolet IS50 Fourier infrared spectrometer (Waltham, MA, USA). The atomic force microscopy (AFM) images were collected by a 5500-type atomic force microscope (Agilent, CA, USA). The X-ray photoelectron spectroscopy (XPS) spectra were taken with an Escalab 250 XI X-ray photoelectron spectrometer (Thermo Fisher, Waltham, MA, USA). The Raman spectra were recorded by an Invia REFLEX laser micro Raman spectrometer (Renishaw, New Mills, UK).

### 2.2. Synthesis of BPNSs

BPNSs were synthesized with the liquid exfoliation method. First of all, we took 20 mg BP crystal powder into 1 mL NMP solution, ground it into small particles with a glass rod and added a further 30 mL of NMP solution when the liquid turned black brown. Then, the upper solution was taken into a beaker. Nitrogen (N_2_) gas was passed through the beaker for 5 min to remove oxygen and reduce the oxidation of BPNSs in the ultrasonic process. Then, the ultrasound was alternated at two frequencies of 40 kHz and 80 kHz for 8 h, respectively. The BP crystal particles were removed by centrifugation at 4000 rpm for 10 min, and the concentrated NMP suspension of BPNSs was prepared by centrifugation at 12,000 rpm for 15 min. The suspension was stored at 4 °C.

### 2.3. Synthesis of Fe^3+^/BPNSs

The synthesis of Fe^3+^/BPNSs was simple. In particular, 200 μL 1 mg·mL^−1^ BPNSs aqueous dispersion and 5 μL FeCl_3_ (0.05 mg·mL^−1^) were mixed and reacted in a metal bath at 25 °C for 30 min.

### 2.4. MTT Assay

Hacat cells were added into a 96-well plate with a density of 1 × 10^5^ cells per well using RPMI Medium 1640 basic (1×) containing 10% FBS and then incubated in a 5% CO_2_ incubator at 37 °C for 24 h. Next, we added BPNS suspensions (100 μL per well) at various concentrations (7.81, 15.63, 31.25, 62.5, 125 μg·mL^−1^) and incubated them in 5% CO_2_ at 37 °C for 24 h. After incubation, we added 100 μL 1.0 mg·mL^−1^ MTT into each well and co-incubated them for 4 h, and then removed the supernatant and 150 μL DMSO was added into wells. Finally, the mixture was shaken for 5 min at room temperature, and then the absorbance at 490 nm was measured.

### 2.5. Electrochemical Characterization

First, the glassy carbon electrode (GCE) was polished with 0.05 μm alumina and cleaned with ethanol and deionized water under ultrasound for 1 min, respectively. GCE was characterized in a 10 mM [Fe(CN)_6_]^3−/4−^ solution containing 0.1 M KCl. After ultrasonic cleaning again, 5 μL 1 mg·mL^−1^ BPNS solution was added to the surface of the electrode after N_2_ drying, and then the electrode was dried in a vacuum dryer for 20 min to obtain the BPNS–GCE electrode. An Fe^3+^/BPNS–GCE electrode was also obtained by the above method.

Electrochemical properties were characterized by cyclic voltammetry (CV) and electrochemical impedance spectroscopy (EIS). Electrochemical reactions both used a three-electrode system, including the modified electrode, platinum wire counter electrode and Ag/AgCl reference electrode. The scanning potential range of CV was 0.30–0.80 V with a scanning rate of 0.05 V·s^−1^, and the electrolyte was PBS (pH = 7.06) buffer solution. EIS detection was carried out under an open circuit potential of 0.23 V (vs. Ag/AgCl) in 10.0 mM [Fe(CN)_6_]^3−/4−^ solution containing 0.1 M KCl. The frequency range was 100 kHz–0.1 Hz.

### 2.6. Detection of Alpha-Fetoprotein

**Scheme 1:**
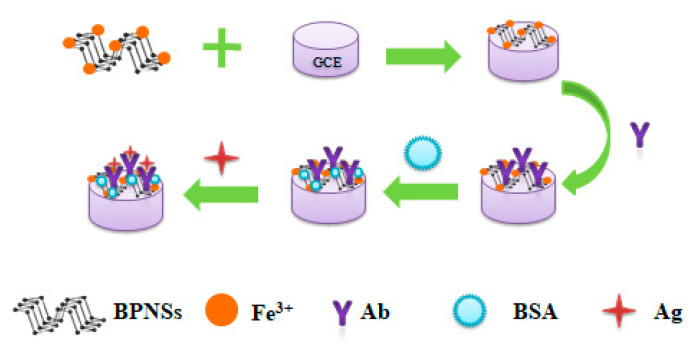
Schematic illustration of the electrochemical immunosensors for the detection of AFP.

As Scheme 1 shows, first, 5 μL 80 μg·mL^−1^ AFP antibody (Ab) was introduced into the Fe^3+^/ BPNS–GCE electrode and it was incubated at 37 °C for 2 h, and then rinsed with PBS 3 times and blown dry with N_2_ to prepare the Ab/Fe^3+^/BPNS–GCE electrode. Next, the BSA/Ab/Fe^3+^/BPNS–GCE electrode was prepared by dripping 5 μL bovine serum protein (1 wt%, BSA) on the surface of the Fe^3+^/BPNS–GCE electrode, incubating it at 37 °C for 0.5 h, rinsing with PBS for 3 times and blowing dry with N_2_. BSA was used to close the excess binding sites on the electrode surface and inhibit non-specific adsorption. Finally, 5 μL AFP antigens (Ag) with different concentrations were introduced into the BSA/Ab/Fe^3+^/BPNS–GCE electrode and incubated at 37 °C for 1 h. AFP antigen and antibody can form specific binding through an immune response. The Ag/BSA/Ab/Fe^3+^/BPNS–GCE electrode was obtained by rinsing with PBS 3 times and blowing dry with N_2_. CV was detected using a three-electrode system. The process of the detection of interferences was the same.

## 3. Results and Discussion

### 3.1. Characterization of BPNSs and Fe^3+^/BPNSs

#### 3.1.1. Structural Analysis

**Figure 1:**
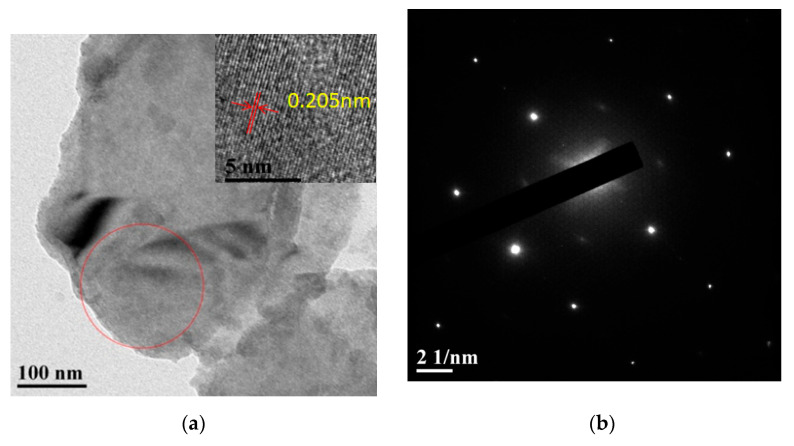

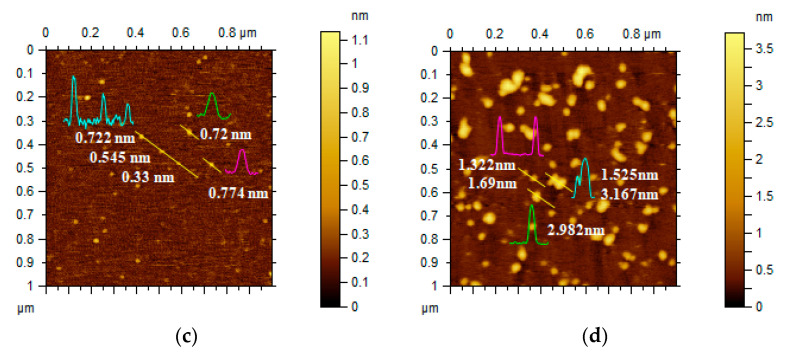
(**a**) TEM and (**b**) SAED images of Fe^3+^/BPNSs; AFM images of (**c**) BPNSs and (**d**) Fe^3+^/BPNSs.

The morphology and structure of Fe^3+^/BPNSs were characterized by transmission electron microscopy (TEM). As shown in Figure 1a, the prepared Fe^3+^/BPNSs have a lamellar structure with relatively thin thickness and fewer layers. The insert image shows that the well-resolved lattice fringe spacing of BPNSs is around 0.205 nm, which corresponds to the Z direction of the BPNSs’ fold structure and belongs to the (022) plane of the BP crystal structure [23]. The selected area electron diffraction (SAED) image (Figure 1b) demonstrates that BPNSs have a regular lattice array, indicating that the prepared BPNS is a single-crystal structure.

According to the EDS diagrams of BPNSs (Figure S1), it can be found that the Fe element is evenly distributed on the surface of BPNSs, which proves the existence of Fe. As NMP was used in the synthesis of BPNSs and the sample was adsorbed onto the conductive adhesive during the characterization process, the C element appeared in the EDS diagrams of BPNSs. In addition, the surfaces of BPNSs and Fe^3+^/BPNSs were characterized by AFM. Figure 1c shows that the thickness of the prepared BPNSs was around 0.330–0.774 nm, which is the thickness of 2–3 layers of BPNSs [24]. The thickness of Fe^3+^/BPNSs was roughly between 1.322 nm and 3.137 nm (Figure 1d), which is significantly larger than that of BPNSs. The results show that after Fe^3+^ is loaded, BPNSs can easily stack and accumulate.

#### 3.1.2. Spectroscopy Analysis

**Figure 2:**
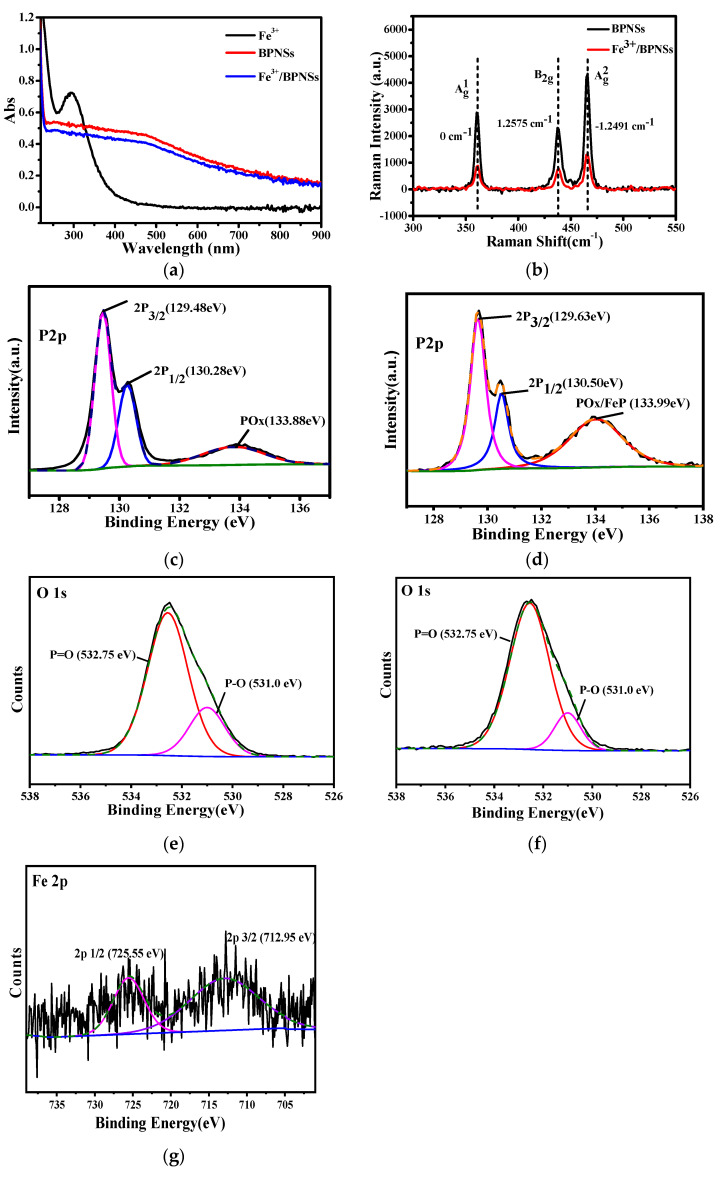
(**a**) UV–vis absorption spectra of BPNSs, Fe^3+^/BPNSs and Fe^3+^. (**b**) Raman spectra of BPNSs and Fe^3+^/BPNSs. High-resolution XPS spectra of P element of (**c**) BPNSs and (**d**) Fe^3+^/BPNSs, O element of (**e**) BPNSs and (**f**) Fe^3+^/BPNSs, (**g**) Fe element of Fe^3+^/BPNSs.

UV–vis absorption spectra were used to verify whether Fe^3+^ was successfully loaded on BPNSs. As shown in Figure 2a, BPNSs have wide absorption in the UV–vis region. After loading by Fe^3+^, the UV–vis absorption intensity of BPNSs decreases. This may be due to the cation–π interaction between Fe^3+^ and BPNSs, which reduces the conjugated structure of BPNSs. The results indicate that Fe^3+^ is successfully loaded on the surface of BPNSs.

The structures of BPNSs and Fe^3+^/BPNSs were also characterized by Raman spectroscopy, and the excitation wavelength was 633 nm. The three Raman characteristic peaks of BPNSs were located at 361, 437 and 466 cm^−1^, respectively. The Ag^1^ peak of Fe^3+^/BPNSs at 361 cm^−1^ is stronger than that of BPNSs, which means that Fe^3+^ is attached on the surface of BPNSs. The Ag^1^/Ag^2^ intensity ratio can highlight the different oxidation states. The Raman spectroscopy (Figure 2b) exhibits that the Ag^1^/Ag^2^ of BPNSs is 0.6701, and the Ag^1^/Ag^2^ of Fe^3+^/BPNSs is 0.7095, both of which are greater than 0.2, indicating that BPNSs and Fe^3+^/BPNSs have a low degree of oxidation and are insensitive to polarization [25]. The intensity ratio is also related to the number of layers in BPNSs; the higher the intensity ratio is, the greater the layer of BPNSs is. As can be seen from Figure 2b, the peak position of Ag^2^ of Fe^3+^/BPNSs blue shifts by 1.2491 cm^−1^, demonstrating that the number of layers of Fe^3+^/BPNSs increases. The above results reveal that the number of layers of BPNSs increases after Fe^3+^ is loaded. This is consistent with the above AFM characterization results.

The chemical composition of Fe^3+^/BPNSs and the chemical states of the elements were analyzed by XPS. BPNSs and Fe^3+^/BPNSs both contain C, P and O elements, and from the element composition ratio of BPNSs and Fe^3+^/BPNSs, it can be seen that the oxygen element content increased from 21.89% to 32.87%. As seen from the high-resolution P 2p spectra of BPNSs and Fe^3+^/BPNSs (Figure 2c,d), they confirm that the oxidation of element P in Fe^3+^/BPNSs is significantly enhanced according to the comparison of the peak area at the highest binding energy in P 2p spectra. It is also found that the binding energy of P 2p in Fe^3+^/BPNSs is larger than that in BPNSs, meaning that Fe^3+^ may bind with P atoms. Compared with the XPS of O 1s (Figure 2e,f), after Fe^3+^ modification, the positions of O 1s peaks do not shift. Due to the low content of Fe^3+^ in Fe^3+^/BPNSs, the Fe 2p peak of Fe^3+^/BPNSs is relatively weaker (Figure 2g). Fe 2p_1/2_ and 2p_3/2_ have larger binding energies than those of FeO_x_ [26]. Therefore, Fe may bind with P to form an Fe–P bond, which is consistent with the reported literature [22].

#### 3.1.3. Electrochemical Analysis of Fe^3+^/BPNSs

**Figure 3:**
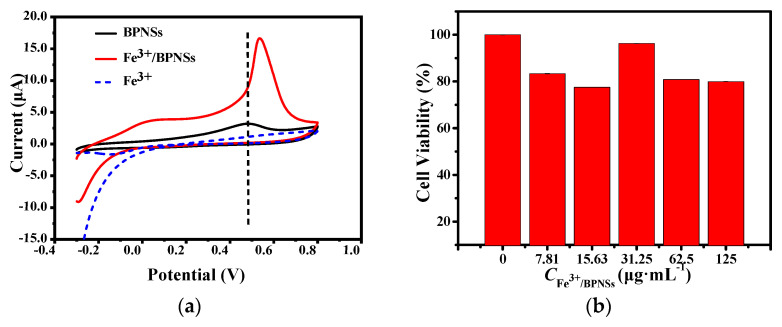
(**a**) CV diagrams of BPNSs and Fe^3+^/BPNSs. (**b**) Cytotoxicity on Hacat cells after treatment with different concentrations of Fe^3+^/BPNSs.

The redox properties of Fe^3+^/BPNSs were characterized by CV. Figure 3 shows that the curve of the CV diagram of BPNSs is asymmetric from top to bottom, indicating that the oxidation of BPNSs is irreversible. However, the peak current and potential of Fe^3+^/BPNSs are significantly changed, indicating that Fe^3+^ is successfully loaded on the surface of BPNSs. The peak current of Fe^3+^/BPNSs is greatly enhanced: it is increased by five times compared with that of BPNSs, revealing that Fe^3+^/BPNSs are more easily oxidized. This is because Fe^3+^ has good catalytic oxidation activity and can catalyze the oxidation of BPNSs [27,28,29].

#### 3.1.4. Cytotoxicity of Fe^3+^/BPNSs

The cytotoxicity of Fe^3+^/BPNSs to Hacat cells was studied by the MTT assay. As shown in Figure 3b, the cell viability remains at 80% after culturing at the concentration of 125 μg·mL^−1^ for 24 h. The result indicates that Fe^3+^/BPNSs have low toxicity and good biocompatibility.

### 3.2. Optimization of Experimental Conditions

**Figure 4:**
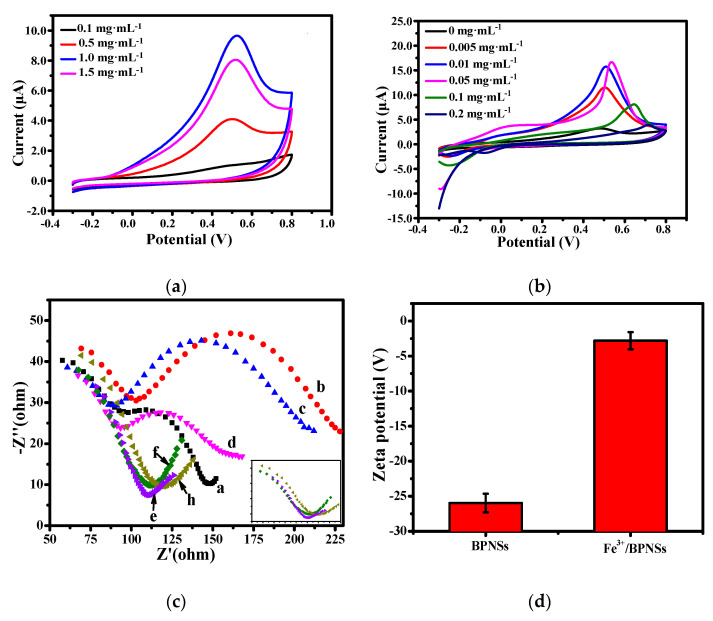

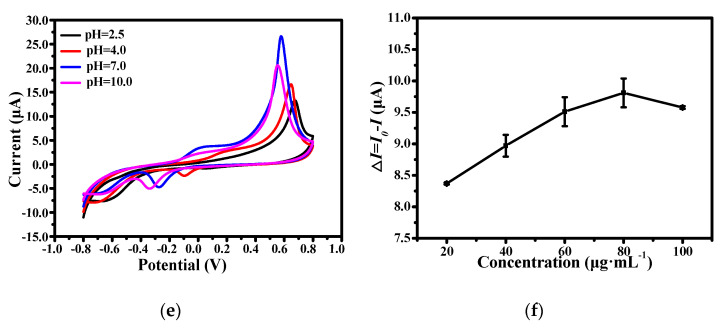
(**a**) CV diagrams of BPNSs with different concentrations, (**b**) CV diagram and (**c**) EIS characterization of Fe^3+^/BPNSs loaded with different concentrations of Fe^3+^. a—0, b—0.005, c—0.01, d—0.05, e—0.1, f—0.2, h—0.4 mg·mL^−1^. (**d**) Zeta potentials of BPNSs and Fe^3+^/BPNSs. CV diagrams of (**e**) Fe^3+^/BPNSs at different pH conditions. (**f**) Current differences at different antibody concentrations.

It can be seen from Figure 4a that the peak current enhances with the increase in the concentration of BPNSs and obtains the maximum value at 1 mg·mL^−1^. As the concentration increases, it may cause the accumulation of BPNSs at a high concentration, which will lead to a decrease in the peak current. Therefore, 1 mg·mL^−1^ BPNSs was selected as the optimal concentration for this electrochemical system.

As shown in Figure 4b, the peak current value of Fe^3+^/BPNSs increases according to the increase in the Fe^3+^ concentration. When the Fe^3+^ concentration is 0.05 mg·mL^−1^, the peak current reaches the largest value. While the concentration of Fe^3+^ continues to increase, the difficulty of oxidation increases, resulting in a decrease in the peak current. In order to prove that the electron transport is blocked, EIS is used to characterize the situation of electron transport. BPNSs have good charge mobility and electrons can transfer quickly on their surface, so the resistance of BPNSs is small (Figure 4c). When Fe^3+^ is loaded on BPNSs, the electrons of P are absorbed by Fe^3+^, and the surface electron transfer is blocked. As the Fe^3+^ concentration increases, the resistance decreases. When the Fe^3+^ concentration is 0.05 mg·mL^−1^, the resistance value is smaller compared with 0.005 mg·mL^−1^ and 0.01 mg·mL^−1^, which is consistent with the result of CV. However, when the concentration is larger than 0.05 mg·mL^−1^, it can be found that the shape of the EIS curve is changed, meaning that the electric circuit of the sensor is varied. Therefore, 0.05 mg·mL^−1^ Fe^3+^ was selected to modify BPNSs. In addition, the absolute value of zeta potential (Figure 4d) reduces after loading Fe^3+^, and the repulsion between layers could decrease, which makes the accumulation of Fe^3+^/BPNSs more serious and hinders the electron transfer on the surface of BPNSs.

The pH value of the PBS buffer solution has different effects on Fe^3+^/BPNSs (Figure 4e). The peak current of Fe^3+^/BPNSs decreases under both acidic and alkaline conditions. When pH = 7, the peak current of Fe^3+^/BPNSs reaches the maximum. Fe^3+^ may produce sediment on the surface of BPNSs at pH > 3.7, resulting in an increase in resistance and a decrease in current. However, since BPNSs are oxidized, Fe^3+^ will coordinate with P. When the pH is 7, the hydroxyl group replaced by the phosphoric acid group is the greatest, followed by pH = 10, and when pH = 4, the hydroxyl group replacement rate is low because there are fewer hydroxyl groups in the solution itself, so the precipitation is less when pH = 7. In an alkaline environment, the dissolution of BPNSs accelerates, so the current drops. Therefore, PBS buffer solution with pH = 7 was selected as the reaction solution.

The extent of current decline reflects the number of antibodies connected on Fe^3+^/BPNSs, so we optimized the concentration of antibodies. As can be seen from Figure 4f, when the antibody concentration is 80 μg·mL^−1^, the current of Fe^3+^/BPNSs drops the most, indicating that this is the maximum load of antibody on Fe^3+^/BPNS–GCE, so the concentration of Ab was set at 80 μg·mL^−1^.

### 3.3. Electrochemical Determination of Alpha-Fetoprotein

**Figure 5:**
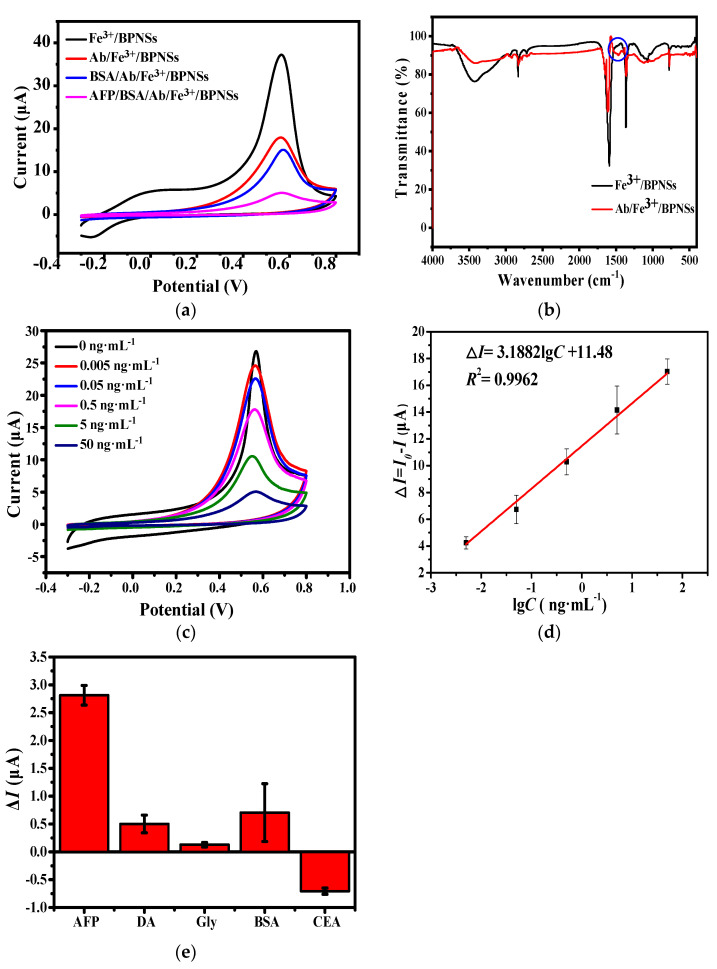
(**a**) CV diagram of the build process and (**b**) Fourier infrared spectroscopy of Fe^3+^/BPNSs and Fe^3+^/BPNSs-Ab. (**c**) CV diagram of the immunosensors at a series of concentrations of AFP. (**d**) Linear relationship between logarithm of antigen concentration and current difference. (**e**) Comparison of current change with different interference factors.

CV was used to characterize the construction process of the sensor, as shown in Figure 5a. Since AFP antibody (80 μg·mL^−1^) is a biological macromolecule, it will attach to Fe^3+^/BPNSs and block electron transfer, resulting in the current reducing. According to the Fourier infrared spectroscopy of Fe^3+^/BPNSs and Ab/Fe^3+^/BPNSs (Figure 5b), there is a peak at 1460 cm^−1^ for Ab/Fe^3+^/ BPNSs, which can be attributed to the combination of the P of Fe^3+^/BPNSs with the C of the antibody [30]. These results indicate that the antibody is effectively connected to Fe^3+^/BPNSs. With the addition of BSA, the steric hindrance increases, so the current decreases. Finally, when the antigen is introduced, the current is further reduced after the antibody is specifically attached to the antigen because proteins act as insulators, which further blocks electron transport. The results show that the immunosensors of AFP are successfully prepared.

The constructed immunosensors were used to detect AFP with different concentrations, which are 0.005 ng·mL^−1^, 0.05 ng·mL^−1^, 0.5 ng·mL^−1^, 5 ng·mL^−1^ and 50 ng·mL^−1^, respectively. Within this concentration range, the logarithmic of AFP concentration (lg*C*) is linear to the current difference between the current of BSA/Ab/Fe^3+^/BPNSs (*I*_0_) and AFP/BSA/Ab/Fe^3+^/BPNSs (*I*) (∆*I*, ∆*I* = *I_0_* − *I*). The linear relationship is ∆*I* (μA) = 3.1882 lg*C* + 11.48 (*R*^2^ = 0.9962) and it has a detection limit (LOD) of 1.2 pg·mL^−1^ (Figure 5c,d), which is determined at S/*n* = 3. The LOD of the designed sensors is sufficient for the detection of AFP in humans. As shown in Table 1, the results show that the electrochemical immunosensors based on Fe^3+^/BPNSs have a lower detection limit for AFP than other immunosensors.

### 3.4. Selectivity of the Electrochemical Immunosensors

Dopamine (DA), carcinoembryonic antigen (CEA) and AFP have similar physical and chemical structures [1], while glycine (Gly) is a coexisting substance of AFP. In addition, bovine serum albumin (BSA) may interfere with the determination of AFP content. Therefore, DA, Gly, CEA and BSA were used to evaluate the specificity of the sensor in this study. As shown in Figure 5e, the current difference of AFP is 5.6 times that of DA, 21.9 times that of Gly and 4 times that of BSA, respectively. The effect of CEA on the immunosensors is contrary to the trend of AFP. Compared with other studies, the designed electrochemical sensors do not have better selectivity. This may be due to the good binding between BPNSs and biomolecules and the large surface area of BPNSs, which may lead to the adsorption of more biomolecules, such as protein and DNA.

## 4. Conclusions

In summary, we successfully prepared BPNSs with a few layers and loaded Fe^3+^ onto the surfaces of BPNSs, which effectively enhanced the electrochemical performance of the BPNSs. The cation–π force is formed because Fe^3+^ absorbs the lone pair electron of the P atom on BPNSs through the vacant orbital. Compared with BPNSs, the oxidation current of Fe^3+^/BPNSs increases by five times, which is beneficial to improve the sensitivity and the detection range of the electrochemical sensors. The electrochemical detection technology based on Fe^3+^/BPNSs is established to realize the highly sensitive detection of AFP. The linear relationship is ∆*I* (μA) = 3.1882 lg*C* + 1.9128 (*R*^2^ = 0.9962) and the LOD is 1.2 pg·mL^−1^. The results show that Fe^3+^/BPNSs can be used as a novel electrochemical sensor for biological detection. However, the electrochemical property of BPNSs has an intrinsic problem wherein BPNSs are easily oxidized to P_x_O_y_ and form phosphate in water, leading to an irreversible electrochemical reaction [36]. Thus, it is difficult to achieve multiple cycles in electrochemistry and the stability is poor for BPNSs. Though the electrochemical sensors based on pristine or metal-ion-modified BPNSs perform poorly in stability, our works explore the influence of metal cations on the electrochemical properties of BPNSs and demonstrate that the direction for the modification of BPNSs is feasible. In future studies, it would be meaningful to study how to slow the oxidation of BPNSs to enhance their stability and decorate BPNSs to improve their selectivity in electrochemical applications. The inherent electrochemistry of BPNSs still means that they have good application prospects in electrochemical sensors.

## Figures and Tables

**Table 1:** Comparison of different methods to detect AFP.

Methods	Linear Range (ng·mL^−1^)	LOD (pg·mL^−1^)	Refs.
Fluorescence	0.01–100	3	[31]
Colorimetry	0–20	35	[32]
Electrochemistry	0.01–100	3	[33]
Electrochemiluminescent immunosensors	0.001–50	3.4	[34]
Photoelectrochemistry	0.1–300	82 (cathode)/54 (anode)	[35]
Electrochemistry	0.005–50	1.2	This work

## Data Availability

Not applicable.

## Supplementary Materials

The following supporting information can be downloaded at: https://www.mdpi.com/article/10.3390/mi13050673/s1. Figure S1. (a) SEM of BPNSs. (b) EDS mapping of C, P, Fe at BPNSs.
