# Supplementary material for: Electrochemical Detection of Alpha-Fetoprotein Based on Black Phosphorus Nanosheets Modification with Iron Ions
Source: Micromachines (Basel). 2022 Apr 26;13(5):673. doi: 10.3390/mi13050673 (PMC9146063; doi:10.3390/mi13050673)
Supplement: Supplementary file 1 [file micromachines-13-00673-s001.zip › micromachines-1646781-supplementary.pdf]

Electrochemical detection of alpha-fetoprotein based on black phosphorus nanosheets modification with iron ions

Yiyan Chen[1], Xiaoping Chen[1,2], Jianwei Lin[1,2], Yafeng Zhuang[1,2], Zhizhong Han[1,2,*], Jinghua Chen[1,2]

[1]School of Pharmacy, Fujian Medical University, Fuzhou 350122, China

[2]Fujian Key Laboratory of Drug Target Discovery and Structural and Functional Research, Fuzhou 350122, China

* Corresponding author:

Email: zzhan@fjmu.edu.cn (Z. Han)

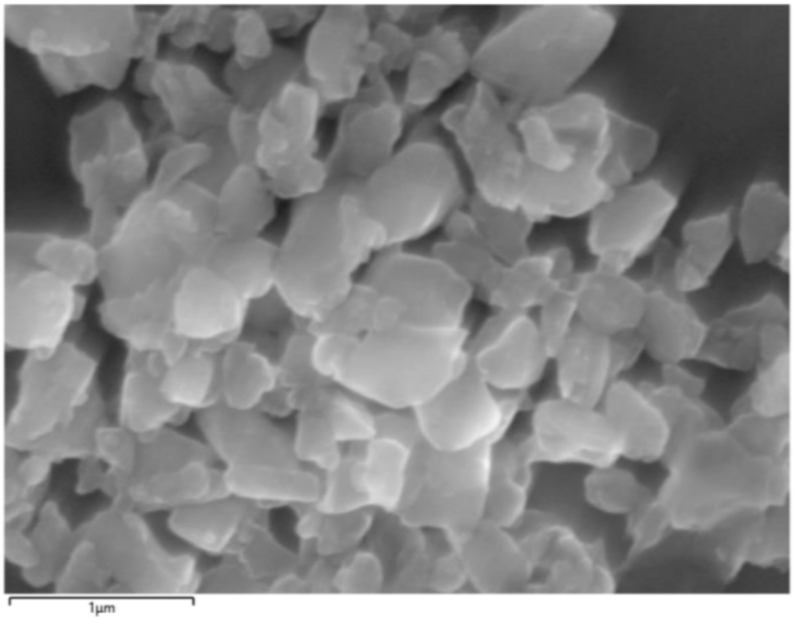

(a)

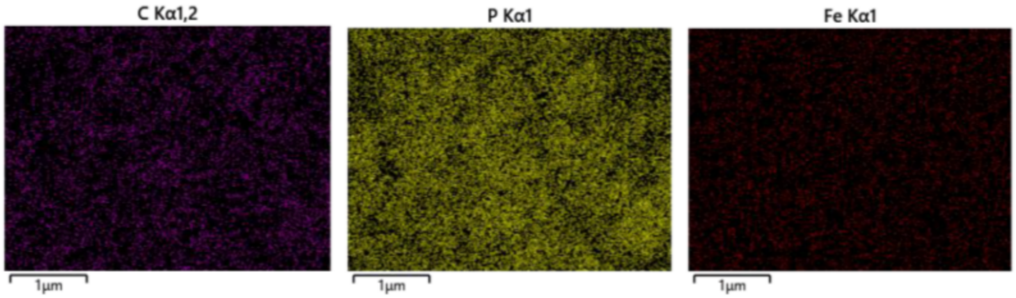

(b)

Figure S1 (a) SEM of BPNSs. (b) EDS mapping of C, P, Fe at BPNSs.
